# Photocatalytic-treated asphalt road in Copenhagen for urban $\text{NO}_{x}$ removal

**DOI:** 10.1007/s10098-022-02441-8

**Published:** 2022-12-09

**Authors:** Lilja Dahl, Henrik Jensen, Alessandro Bigi, Grazia Ghermandi

**Affiliations:** 1 Department of Engineering “Enzo Ferrari” (DIEF), University of Modena and Reggio Emilia (UniMoRe), Via P. Vivarelli 10, 41125 Modena, Italy; 2 Photocat A/S, Langebjerg 4, 4000 Roskilde, Denmark

**Keywords:** Air quality, Nitrogen dioxide, Photocatalysis, Titanium dioxide, Clean-air technology

## Abstract

Atmospheric nitrogen oxides ($\hbox {NO}_{x} = \hbox {NO}+\hbox {NO}_{2}$) are key pollutants and short-lived climate forcers contributing to acid rain, photochemical smog, aerosol formation and climate change. Exposure to nitrogen dioxide ($\hbox {NO}_{2}$) emitted mainly from transportation, causes adverse health effects associated with respiratory illnesses and increased mortality even at low concentration. Application of titanium dioxide ($\hbox {TiO}_{2}$)-based photocatalysis in urban environment is a new air cleaning solution, activated by sunlight and water vapour to produce OH radicals, able to remove $\hbox {NO}_{x}$ and other pollutants from the planetary boundary layer. This study is a large-scale evaluation of $\hbox {NO}_{x}$ removal efficiency at a near-road environment with applied photocatalytic NOxOFF™ technology on an urban road west of Copenhagen, thus supporting local municipality in meeting their clean-air Agenda 2030. The photocatalytic NOxOFF™ granulate containing $\hbox {TiO}_{2}$ nanoparticles was applied on an asphalt road in July 2020 and ambient $\hbox {NO}_{x}$ was measured during a six-month monitoring campaign. It is the first $\hbox {NO}_{x}$ monitoring campaign carried out at this road and specific efforts have been devoted to evaluate the reduction in ambient $\hbox {NO}_{x}$ levels with NOxOFF™-treated asphalt. Several methods were used to evaluate the photocatalytic effect, taking into account analysis limitations such as the short reference period prior to application and the highly uncertain measurement period during which SARS-CoV-2 lockdown measures impacted air quality. There was no statistically significant difference in $\hbox {NO}_{x}$ concentrations between the reference period and the photocatalytic active period and NO removal efficiency resulted in $-\,0.17$ (± 1.27). An upper limit removal of 17.5% $\hbox {NO}_{x}$ was estimated using a kinetic tunnel model. While $\hbox {NO}_{2}$ comparison with COPERT V street traffic model projection was roughly estimated to decrease by 39% (± 38%), although this estimate is subject to high uncertainty. The observed annual mean $\hbox {NO}_{2}$ concentration complies with Frederiksberg clean-air Agenda 2030 and air quality standards.

**Graphical abstract:**

A graphical abstract illustrating the air cleaning properties of $\hbox{TiO}_{2}$-based photocatalytic-treated asphalt 
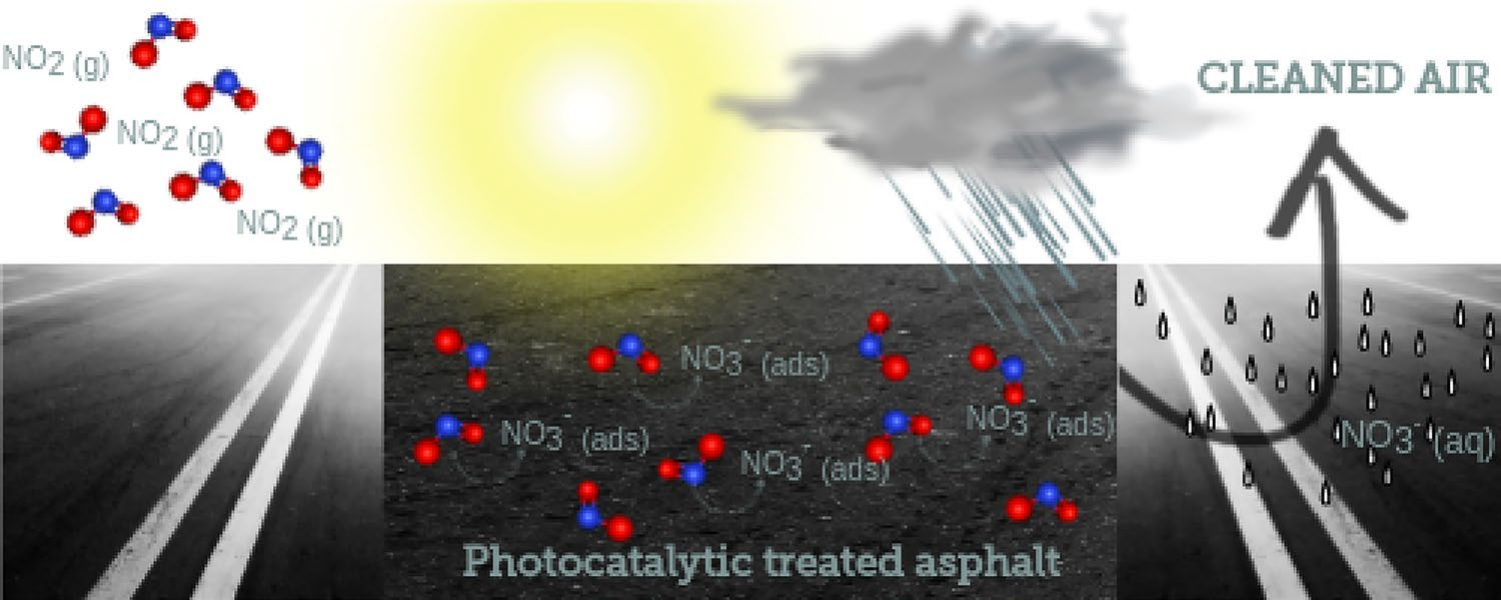

## Introduction

Along with continuous urbanisation, air pollution plumes from cities are dominated by anthropogenic emissions, causing poor air quality (AQ) levels in urban areas and detrimental impacts on citizens health and ecosystem (Öztürk et al. 2018). Exposure to ambient and indoor air pollution is estimated by World Health Organization (WHO) to cause more than 7 million premature deaths globally every year (WHO et al. 2021). In Europe, 417k premature deaths were directly linked to long-term exposure of ambient fine particulate matter ($\hbox {PM}_{2.5}$), 55k associated with nitrogen dioxide ($\hbox {NO}_{2}$) exposure and 20.6k related to tropospheric ozone ($\hbox {O}_{3}$) exposure in 2018 (González Ortiz et al. 2020).

In the urban environment, $\hbox {NO}_{x}$ (NO  + $\hbox {NO}_{2}$) is considered one of the predominant atmospheric pollutant, produced during high temperature combustion processes from both mobile and stationary sources. The largest source of $\hbox {NO}_{x}$ in Europe is the road transport sector, contributing to 39% of all EU-28 countries $\hbox {NO}_{x}$ emission in 2017 (González Ortiz et al. 2019). The direct health impacts of $\hbox {NO}_{x}$ is related to the secondary pollutant $\hbox {NO}_{2}$, a toxic red brown gas that irritates lungs and causes respiratory infection at high concentration (Khreis et al. 2020). A review and a meta-study by the UK Committee On the Medical Effects of Air Pollutants (https://www.gov.uk/government/collections/comeap-reports) concludes that health effects of long-term $\hbox {NO}_{2}$ exposure is associated with increased mortality, although it remains challenging to separate the synergistic effects of $\hbox {NO}_{2}$ with other pollutants. Furthermore, epidemiological evidence suggests that an exposure to only 5 $\upmu \text {g}\,\text {m}^{-3}$ annual average is associated with increased mortality (COMEAP 2018). Consequently, WHO have recently updated their AQ guidelines and lower the average limit of $\hbox {NO}_{2}$ to 10 $\upmu \text {g}\,\text {m}^{-3}$ (1/4 of what was recommended in 2005) and 25 $\upmu \text {g}\,\text {m}^{-3}$ over the year and day, respectively, based on a systematic review of latest medical evidence (WHO et al. 2021).

**Fig. 1:**
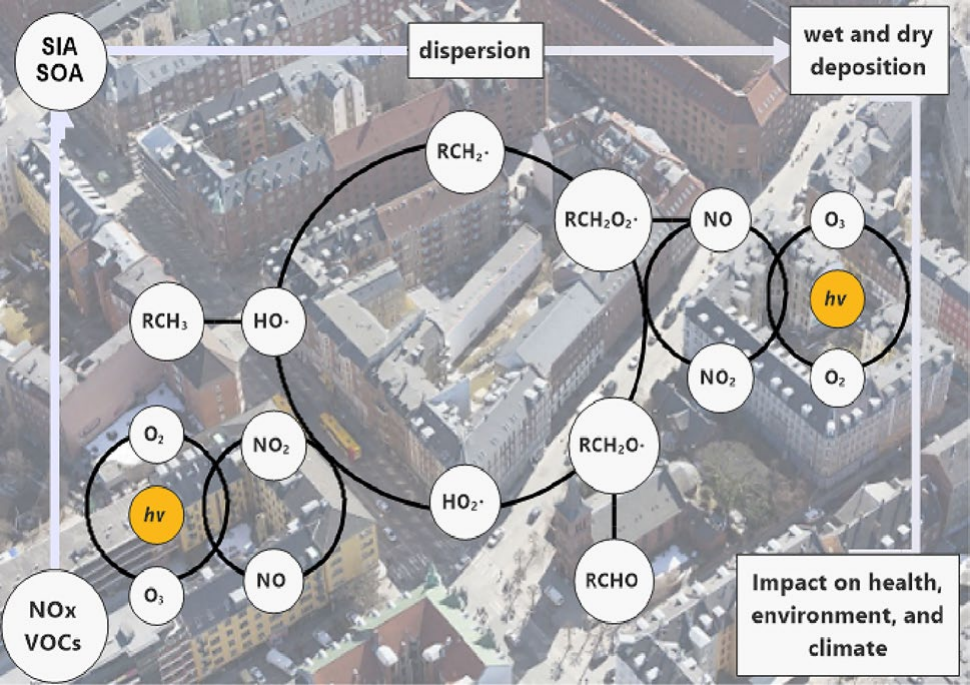
The general reaction mechanism of $\hbox {NO}_{x}$ and VOCs oxidation by OH radical and $\hbox {O}_{2}$ in the troposphere. SIA and SOA refers to Secondary Inorganic Aerosol and Secondary Organic Aerosol. Adapted from Harnung and Johnson (2012)

$\hbox {NO}_{2}$ lifetime in the troposphere is mainly controlled by solar radiation, OH, peroxide radicals and $\hbox {O}_{3}$. It is a moderately long-lived species with a lifetime of 1 day and can be transported on a local spatial scale: given a vertical mixing of the troposphere of 1 week, $\hbox {NO}_{x}$ is not well mixed vertically throughout the troposphere (Seinfeld and Pandis 2016). $\hbox {NO}_{x}$ and volatile organic compounds (VOCs) under sunlight can form photochemical smog and decrease visibility according to the reaction pathway shown in Fig. 1. Moreover, $\hbox {NO}_{2}$ can be converted to nitrous and nitric acid, which are further neutralised by ammonia to nitrite and nitrate salts, generally referred as Secondary Inorganic Aerosol (SIA) and representing a large mass fraction of atmospheric $\hbox {PM}_{2.5}$. SIA has a longer lifetime in the atmosphere and can build-up especially under specific meteorological conditions, e.g. temperature inversions, leading to significant health effects (González Ortiz et al. 2019).

European cities must meet the EU AQ  directive limit of 40 $\upmu \text {g}\,\text {m}^{-3}$ annual average $\hbox {NO}_{2}$ (EEA 2021). However, 10% of all ambient air monitoring stations in Europe showed values above the $\hbox {NO}_{2}$ yearly limit in 2017 (some cities reported values twice the limit), of which 86% of these stations were located in a roadside environment (Degrauewe et al. 2019).

Starting from 2012 (EU Regulation 459/2012) the EU introduced increasingly stringent emission standards for passenger cars and light duty vehicles exhaust emissions. These regulations originate from the “dieselgate” scandal regarding some defeat devices active in diesel-fuelled cars under regulatory test cycles, which led to $\hbox {NO}_{x}$ emissions largely above the Euro 6 approval limit of 80 mg km$^{-1}$ (Franco et al. 2014; Khreis et al. 2020). With the aim of minimising the gap between the vehicle emissions during laboratory tests and during road driving, two main changes entered into force since 2017 for vehicles type-approval: the use of the Worldwide harmonized Light vehicle Test Procedures (WLTP) at the dynamometer and the on-road testing procedure to estimate the Real Drive Emissions (RDE) (Williams and Minjares 2016).

Since the introduction of catalytic converters in vehicles in 1992, $\hbox {NO}_{x}$ levels in the troposphere experienced a steep decrease in western Europe (Carslaw et al. 2019). The development of efficient emission control technologies and Euro 1-6/I-VI norms have been one of the most valuable ways to improve urban air quality. In order to meet the emission standard, most of diesel vehicles are equipped with a Selective Catalytic Reduction (SCR) catalyst to control the engine-out emissions of $\hbox {NO}_{x}$ by adding urea as a reducing agent (Ayodhya and Narayanappa 2018). Currently for petrol vehicles the main $\hbox {NO}_{x}$ aftertreatment system is the Three-Way Catalysts (TWC), containing platinum group metals reducing $\hbox {NO}_{x}$ to $\hbox {N}_{2}$ as well as oxidising VOCs to carbon dioxide (Shelef and McCabe 2000). With the renewal of vehicular fleets across Europe, and the spread of newer aftertreatment devices, the $\hbox {NO}_{2}$/$\hbox {NO}_{x}$ levelled off since a decade, leading to a faster-than-expect reduction in $\hbox {NO}_{2}$ levels in Europe (Grange et al. 2017).

Further development of $\hbox {NO}_{x}$ aftertreatment systems to meet upcoming Euro 7/VII and future standards for fast developing vehicle fleets is expected to be both costly and technologically challenging (Khreis et al. 2020). It is therefore necessary to continue implementing the most effective AQ regulation, also at a municipality level in order to approach zero pollution in Europe as well as meeting the Sustainable Development Goals (SDGs). An integrated mitigation approach addressing both emission control technologies and new innovative air cleaning solutions can aid municipalities to reach their clean-air objectives and improve urban AQ within the city.

Titanium dioxide ($\hbox {TiO}_{2}$) photocatalysis, is used for the oxidation of atmospheric pollutants, i.e. $\hbox {NO}_{x}$, VOCs, $\hbox {SO}_{x}$, and $\hbox {O}_{3}$ (Chen et al. 2012; Tsang et al. 2019). A process initiated by ultraviolet (UV) radiation (with wavelength shorter than 388 nm) and atmospheric water vapour which, catalysed by $\hbox {TiO}_{2}$, produce OH radicals, able to oxidise $\hbox {NO}_{2}$ to nitric acid which finally undergoes atmospheric wet deposition in the form of nitrate ion ($\text {NO}_3^-$):

1 $$\begin{aligned}{} & {} \text {NO}_{2}(\text {g})+\text {HO}\cdot(\text {g}) \xrightarrow {{\hbox {TiO}_{2}}} \text {HNO}_{3}(\text {g}) \end{aligned}$$

2 $$\begin{aligned}{} & {} \text {HNO}_{3}(\text {g}) \rightleftarrows \text {HNO}_{3}(\text {aq}) \end{aligned}$$

3 $$\begin{aligned}{} & {} \text {HNO}_{3}(\text {aq}) \rightleftarrows \text {NO}_{3}^{-} {(\text {aq})} +\text H^{+} {(\text {aq})} \end{aligned}$$

The photocatalytic effect of $\hbox {TiO}_{2}$ was first published by Renz (1921) a century ago and its mechanism by Fujishima and Honda (1972) five decades later, where $\hbox {TiO}_{2}$ was irradiated with UV light splitting water into hydrogen and oxygen. In more recent years, $\hbox {TiO}_{2}$-based photocatalysis has been used to remove both indoor and outdoor pollutants, fungi as well as viruses (Zhao and Yang 2003; Foster et al. 2011; Chen et al. 2012). It has been applied on asphalt roads, pavements, buildings and roofs among others and demonstrated to significantly remove $\hbox {NO}_{x}$ from ambient air, as well as to have self-cleaning properties (Benedix et al. 2000; Laufs et al. 2010; Martinez et al. 2011; Cardellicchio 2020; Pedersen et al. 2021). Several previous studies tested $\hbox {TiO}_{2}$-based photocatalytic surfaces for their removal rate of atmospheric pollutants at mid latitudes. For instance, Folli et al. (2015) observed up to a 22% monthly reduction of NO during summer, using an application of $\hbox {TiO}_{2}$-based pavement in a 200 m-long stretch of a sidewalk in Copenhagen (Denmark). Kleffmann (2016) later criticised this paper for overestimating $\hbox {NO}_{x}$ abatement, as he theoretically estimated an upper limit removal of only 0.8% NO, using a simple tunnel model assuming first order uptake kinetics. Kleffmann model was also used to estimate $\hbox {NO}_{x}$ abatement in Leopold II tunnel in Brussels after applying photocatalytic coating of a 160 m section, resulting in a theoretical worst case scenario of $\le$2% $\hbox {NO}_{x}$ removal at 4 W m$^{-2}$ UVA irradiance, although theoretically a highly photocatalytic active material (i.e. no deactivation and dirt) with > 10 W m$^{-2}$ irradiance has a potential of > 20% NO removal (Gallus et al. 2015). A more recent European Life project called LIFE PHOTOSCALING, applied photocatalytic coating to 4200 m$^{2}$ pavement on a street in Madrid and achieved an average $\hbox {NO}_{2}$ reduction of 28% for the first three months when compared with a reference street (Castellote 2019). Two real-world photocatalytic experiments conducted in Roskilde municipality and Copenhagen Airport in Denmark for the period 2012–2016, tested the photocatalytic $\hbox {NO}_{x}$ removal capacity of the NOxOFF™ air-cleaning technology from Photocat A/S, after its application on asphalt and concrete tiles in the parking lots. The three year long in situ study at Roskilde demonstrated that NOxOFF™ applied on asphalt was estimated to remove 13.8 g m$^{-2}$ $\hbox {NO}_{x}$ per year, while photocatalytic-treated concrete tiles at Copenhagen airport resulted in a reduction of 12% in ambient $\hbox {NO}_{x}$ levels (Pedersen and Jensen 2021). The photocatalytic coating has a high stability throughout the years, resulting in a potential of approx. 7 kg $\hbox {NO}_{x}$ removed for every 50 m$^{2}$-treated asphalt given that 10 year is the considered lifetime of the photocatalytic coating (Pedersen and Jensen 2021). The societal health cost related to local $\hbox {NO}_{2}$ emissions from traffic in Copenhagen (Denmark) and regional/European $\hbox {NO}_{x}$ emissions is calculated by regional and urban models to have a health cost of approx. 80.7€ per kg $\hbox {NO}_{2}$ (Andersen et al. 2019). The $\hbox {TiO}_{2}$-based NOxOFF™ catalyst is therefore considered a cost effective method to improve air quality in cities and has a payback time of only 2–3.5 years (Pedersen and Jensen 2021).

The current study focuses on an experimental test of the removal efficiency of a $\hbox {TiO}_{2}$-based photocatalytic treated asphalt road in Frederiksberg municipality located in the greater Copenhagen area (Denmark) in 2020. This municipality is implementing actions to limit atmospheric pollution and recently presented a new clean-air agenda aiming to meet the EU AQ directives by the application of several methods, including photocatalytic technology on asphalt roads and pavements to remove $\hbox {NO}_{x}$ from the ambient air.

## Materials and methods

**Fig. 2:**
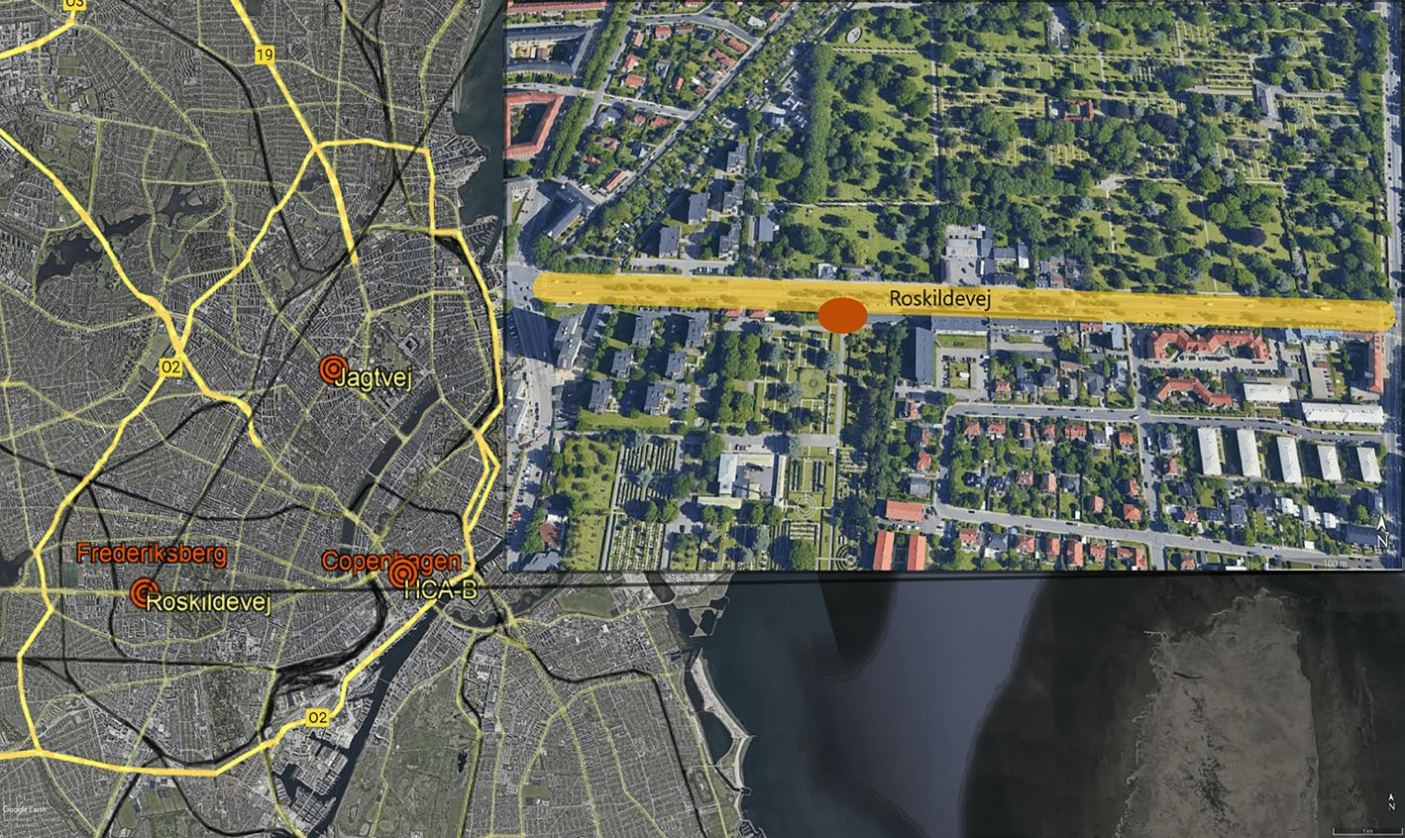
A map of Frederiksberg municipality and Copenhagen municipality (yellow lines indicate major roads and black lines indicate S-train and metro). Roskildevej with applied photocatalytic granulates is highlighted in yellow and the air quality monitoring and weather station site is pointed out in orange

$\hbox {TiO}_{2}$-based photocatalytic asphalt using NOxOFF™ technology was used over approx. 3.5k m$^{2}$ of an urban stretch of the Roskildevej road in Frederiksberg municipality (Fig. 2).

This asphalt, supplied by Photocat A/S and delivered as ultra-high performance concrete containing photocatalytic material made of $\hbox {TiO}_{2}$ nanoparticles in the anatase form, was applied by the asphalt production company Colas A/S on 24 July 2020. The photocatalytic granulates were sprinkled on hot asphalt immediately after the asphalt was paved and had a temperature above 130 $^\circ \text {C}$ and before the asphalt was compacted. The granulates were sprinkled by a ‘chips spreader’ mounted on a conventional asphalt compacter. After application of the photocatalytic granulates, they were compacted together with the asphalt. The photocatalytic granulates are 1–3 mm and the loading was approx. 1.3 kg m$^{-2}$.

An atmospheric monitoring campaign was performed in proximity to the road where this asphalt was applied from 15th of June, i.e. before the application of the photocatalytic asphalt, until 31st of December 2020. The campaign included a $\hbox {NO}_{x}$/NO monitor at the sidewalk approx. 1.5 m from the road and at 2.10 m height and an automatic weather station (AWS) was installed at the sidewalk approx. 4 m from the road at approx. 4 m height. C.K. Environment A/S was responsible for the calibration, collection and QA/QC of these monitors. The statistical analysis was carried out after data validation using the open source programming software R (R Core Team 2020), supported by the Openair package, for state-of-the-art air quality data analysis (Carslaw and Ropkins 2012).

NO and $\hbox {NO}_{x}$ were measured at a 10 seconds time resolution by an ECOPhysics chemiluminescent monitor (Eco Physics CLD 66, ECO PHYSICS AG, Switzerland). $\hbox {NO}_{2}$ was computed by the subtraction of observed NO from $\hbox {NO}_{x}$. The chemiluminescence system is measuring NO from the ambient air by reacting NO with excess $\hbox {O}_{3}$: this produces excited $\hbox {NO}_{2}$, emitting radiation during its quenching, which is then used as an indirect measure of NO concentration. $\hbox {NO}_{x}$ is measured by reducing $\hbox {NO}_{2}$ into NO by the means of a molybdenum catalytic converter upstream of the reaction chamber. This type of converter is known for being prone to overestimate of $\hbox {NO}_{x}$, and therefore of $\hbox {NO}_{2}$, due to the reduction of other oxidised nitrogen gas compounds if present, e.g. $\hbox {HNO}_{2}$, $\hbox {HNO}_{3}$, peroxyacetylnitrate and other organic nitrate species (e.g. Villena et al. 2012; Dunlea et al. 2007). The AWS was measuring meteorological parameters, e.g. temperature, wind speed, wind direction, relative humidity, atmospheric pressure and incoming solar radiation. Since UV radiation is needed to activate the photocatalytic process, the incoming solar UV radiance was derived from the local measurement of global radiation, by computing the share of UV in global solar radiation based on ERA5 reanalysis products (Hersbach et al. 2018) for the study site during the investigated period.

Owing to technical issues, chemiluminescence detection failed to extract valuable data for several days in August and all invalid data such as calibration episodes (at 1500 ppb NO) and abnormal spikes were removed accordingly. Raw data contained some negative NO values that were set to 1/3 of the detection limit of 0.5 ppb. Thereafter, a rolling median with a window of 3 was applied to smooth the 1-min data. Finally, the appropriate conversion factor for converting ppb to $\upmu \text {g}\,\text {m}^{-3}$ is based on European Commission standards at 20°C assuming an atmospheric pressure of 1 atm (Middleton et al. 2007).

### Assessment methods for the $\hbox {NO}_{x}$ removal

Three methods were used to assessed the $\hbox {NO}_{x}$ removal by the photocatalytic surface. Two relies directly on the difference in $\hbox {NO}_{x}$ observations collected before and after the installation of the asphalt, while the third method is based on the application of a chemical reaction model “Tunnel photo-red” built to estimate the photocatalytic remediation of $\hbox {NO}_{x}$ in road tunnels (Gallus et al. 2015).

The former method relies on a reference period of 30 days, between 15 June and 24 July; due to its short length, the reference period was compared to a period of the same length, featured by maximum UV radiation, i.e. between 12PM and 2PM GMT+1. NO concentration during this latter period, defined as the Photocatalytic Active Period (PAP), when the photocatalytic removal is expected to be maximum, was compared with the NO during the reference period for the same hour range, using equation 4 (Folli et al. 2015).

4 $$\begin{aligned} \eta = \frac{[\text {NO}]_{\textrm{reference}}-[\text {NO}]_{\text {PAP}}}{[\text {NO}]_{\textrm{reference}}} \end{aligned}$$

The direct comparison of the reference and the PAP periods is partly hampered by their short length and by the national lockdown enforced in Denmark until the end of June 2020, lockdown due to the SARS-CoV-2 pandemic and which caused many structural changes. Remote work and travel restrictions produced a drastic drop in $\hbox {NO}_{2}$ emissions (approx. 35% of $\hbox {NO}_{2}$ emissions worldwide in 2020 according to Masson-Delmotte et al. (2021)), leading to a large decrease in atmospheric $\hbox {NO}_{2}$ levels, including European pollution hot spots, e.g. in Milan and Barcelona, where more than 50% $\hbox {NO}_{2}$ reduction was observed (González Ortiz et al. 2020). Since July 2020 and to the beginning of August 2020, Denmark reopened education, work and other activities and went gradually into business as usual conditions until the 9th of December, when 38 municipalities (including Frederiksberg) partially closed until the 25th of December, when a full lockdown was finally enforced again (TV2 et al. 2020; Frederiksen 2020a, b).

In order to minimise the bias in traffic emissions induced by the lockdown, and to a smaller extent by the vacation period in July, a second method to estimate the $\hbox {NO}_{x}$ abatement by the photocatalytic asphalt is based on the comparison of atmospheric levels in Roskildevej to levels in two regulatory air quality monitoring sites in Copenhagen, Jagtvej and H.C. Andersens Boulevard (hereafter HCA-B). The second method is therefore based on the $\hbox {NO}_{x}$/$\hbox {NO}_{x}$ ratio comparison between Roskildevej and both Jagtvej and HCA-B, which are urban traffic sites, with the latter sited in the city centre of Copenhagen, on a street with larger traffic than Jagtvej and Roskildevej. While Jagtvej, with about a daily traffic flow of 22k vehicles per day, has similar traffic conditions to Roskildevej, of about 17.9k vehicles per day (personal communication with Frederiksberg municipality). The $\hbox {NO}_{x}$ ratio during the reference period and the PAP were compared for statistically significant differences in their distribution using the Kruskal–Wallis and Wilcoxon–Mann–Whitney nonparametric statistical tests (Sprent and Smeeton 2001).

The last method is an estimation of the upper limit photocatalytic remediation of $\hbox {NO}_{x}$ and it is based on first order reaction kinetics using the tunnel model tool developed by the European project PhotoPaq (Photocatalytic Remediation Processes on Air Quality) and recommended by Kleffmann (2016). The uptake coefficient of NO is calculated by this model to be $1.73\cdot 10^{-5}$ g following the standard ISO 22197-1 laboratory tests of 18% reduction using AirClean^©^ granulates developed and measured by European funded NaHITAs project. Same photocatalytic granulates (named NOxOFF^TM^) were applied on Roskildevej and F.C. Nüdling Betonelemente verified its photocatalytic activity both in laboratory, at a real-life street canyon and at a real field site in China. The street canyon variables and geometry that were used to estimate the averaged upper limit $\hbox {NO}_{x}$ remediation for the entire period after photocatalytic application are: 6.6 W m$^{-2}$ UV irradiance (UV-A accounts for 95% thereof (IARC 2012)); 1.3 m s$^{-1}$ mean wind speed; 84.6% mean relative humidity; 5 m road width (only one lane was removed and applied with photocatalytic asphalt); 2.10 m measurement height and 705 m length of active section.

A final assessment of the $\hbox {NO}_{2}$ variability at Roskildevej was performed by its comparison with urban-scale model simulation of annual $\hbox {NO}_{2}$ levels for Frederiksberg municipality. These simulations were performed by the Danish Centre for Environment and Energy (DCE) to analyse the atmospheric levels in 2018 using a chain of three models: the Danish Eulerian Hemispheric Model (DEHM, Christensen 1997), the Urban Background Model (UBM, Kumar et al. 2019) and the Operational Street Pollution Model (OSPM, Berkowicz 2000). Uncertainties reported for the annual average $\hbox {NO}_{2}$ estimated by this model chain in 2019 were within 0% and 25% (DCE 2021). This model setup, besides providing estimates of regulatory pollutants in 2018 at a 1 km $\times$1 km horizontal resolution in urban background conditions, it was used to produce projection to 2020 based on expected traffic emissions estimated by the emission model COPERT V (Jensen et al. 2020).

## Results and discussion

Table 1 lists the daily mean (± standard deviation) for $\hbox {NO}_{2}$, $\hbox {NO}_{x}$ and their ratio at Roskildevej (representative of Frederiksberg urban background) along with Jagtvej and HCA-B during the reference period (15th of June–24th of July), the PAP and for each month over the period September–December. The overall mean for $\hbox {NO}_{2}$ over the study period resulted in 12.5 (± 5.9) $\upmu \text {g}\,\text {m}^{-3}$, 21.6 (± 9.9) $\upmu \text {g}\,\text {m}^{-3}$ and 28.7 (± 11.9) $\upmu \text {g}\,\text {m}^{-3}$ for Roskildevej, Jagtvej and HCA-B, respectively. These values, although representative of only 6 months, are lower than modelling results: these latter estimated that the mean annual $\hbox {NO}_{2}$ concentration in Frederiksberg ranged from 21–29 $\upmu \text {g}\,\text {m}^{-3}$ in 2018 (Jensen et al. 2020) and projected a decrease from 29 $\upmu \text {g}\,\text {m}^{-3}$ in 2016 to 24 $\upmu \text {g}\,\text {m}^{-3}$ in 2020 for the Copenhagen area (including Frederiksberg). The projections of $\hbox {NO}_{2}$ for 2020 were two times greater than observed, although it did not consider SARS-CoV-2 restrictions on traffic and on air pollution levels. The simulation results are affected by uncertainties, e.g. the horizontal resolution of UBM, which focuses on background concentration, is coarse and a single cell is larger than the area of the Frederiksberg municipality (Jensen et al. 2020).

**Fig. 3:**
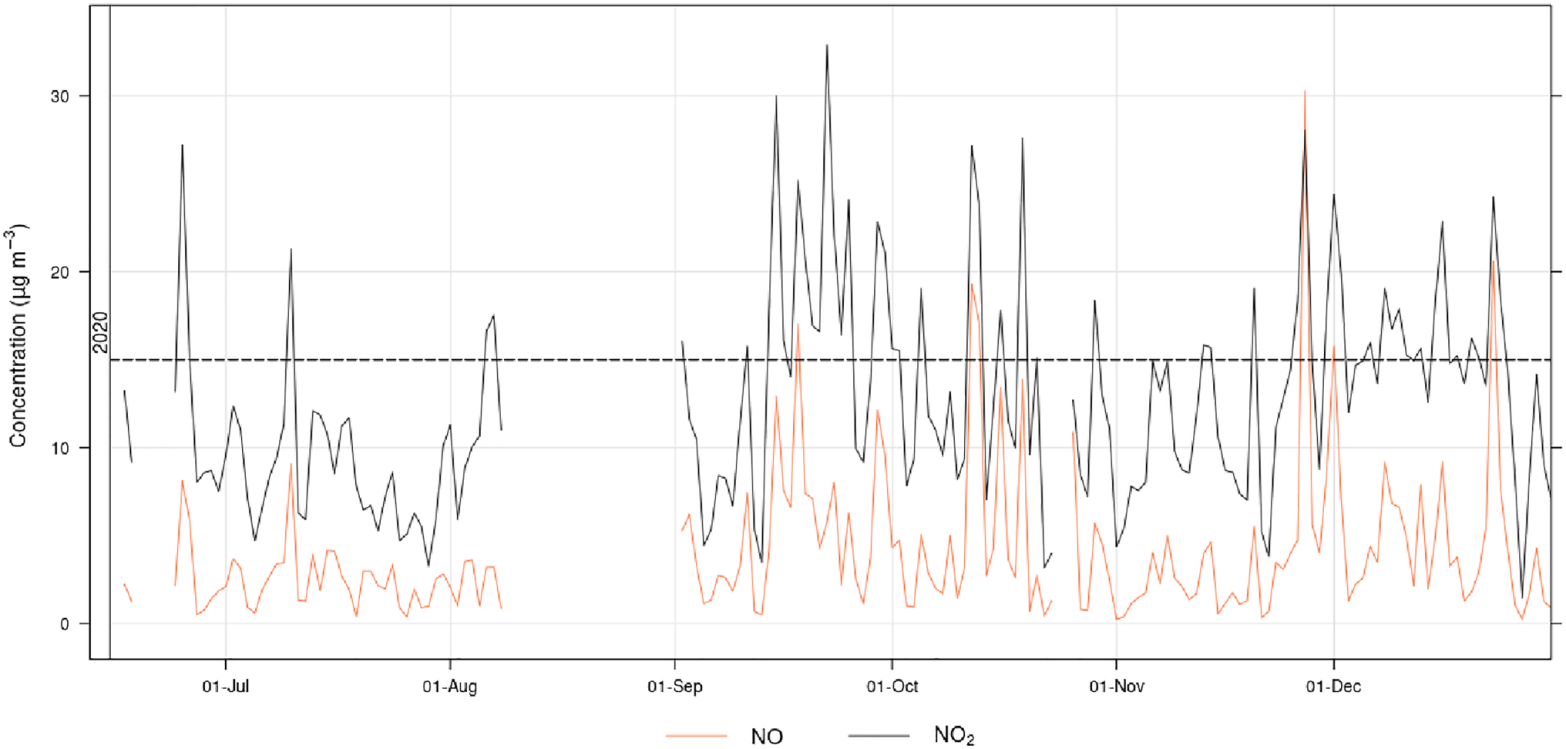
Daily averaged time series of available NO and $\hbox {NO}_{2}$ data in the period June 15th–December 2020. The dashed line indicates the concentration goal set by the Frederiksberg local administration

The $\hbox {NO}_{2}$:$\hbox {NO}_{x}$ ratio is used to understand the chemistry and conversion of NO and $\hbox {NO}_{2}$. This fraction varies widely depending on the site and the traffic characteristics and increases usually from near-road site to rural site. A relatively recent study have reported an average $\hbox {NO}_{2}$:$\hbox {NO}_{x}$ ratio ranging from 0.25 to 0.35 near roads in Las Vegas (Richmond-Bryant et al. 2017). Over the period 2012–2018, ratios ranging between 0.35 − 0.45 were observed at two road sites in Hong Kong (Huang et al. 2020). A higher hourly mean $\hbox {NO}_{2}$:$\hbox {NO}_{x}$ ratio of 0.72 (± 0.1) was observed at Roskildevej after applied photocatalytic asphalt treatment in the PAP period, while at the urban traffic sites in Copenhagen, HCA-B and Jagtvej, the $\hbox {NO}_{2}$:$\hbox {NO}_{x}$ ratio was 0.53–0.55 (± 0.1) during the same period. A previous Europe-wide study (Cyrys et al. 2012) found for Copenhagen a ratio of 0.72 for urban background and a range of 0.42–0.73 for urban traffic sites in 2009–2010, confirming that the Roskildevej site, during the monitoring campaign, was well representative of urban background conditions.

**Table 1:** Daily mean (± standard deviation) for NO, $\hbox {NO}_{2}$ and $\hbox {NO}_{2}$:$\hbox {NO}_{x}$ at Roskildevej, Jagtvej and HCA-B during the Reference period (June 15th – July 24th), the Photocatalytic Active Period (PAP, see text for details) and the months of September, October, November and December

Period	Roskildevej			Jagtvej			HCA-B		
	NO	$$\hbox {NO}_{2}$$	$$\hbox {NO}_{2}$$\$$\hbox {NO}_{x}$$	$$\hbox {NO}_{2}$$	$$\hbox {NO}_{x}$$	$$\hbox {NO}_{2}$$\$$\hbox {NO}_{x}$$	$$\hbox {NO}_{2}$$	$$\hbox {NO}_{x}$$	$$\hbox {NO}_{2}$$\$$\hbox {NO}_{x}$$
Reference	2.4 ± 1.6	8.6 ± 4.4	0.68 ± 0.1	20.8 ± 15.1	39.2 ± 30.1	0.55 ± 0.1	27 ± 12.5	53 ± 24	0.51 ± 0.06
PAP	2.8 ± 2.1	12 ± 8.0	0.72 ± 0.1	20.8 ± 13.8	38.8 ± 25.8	0.55 ± 0.1	30.7 ± 11.9	58.3 ± 19.2	0.53 ± 0.09
September	5.2 ± 10.4	14. 6 ± 11.9	0.71 ± 0.2	22.9 ± 17.2	41.8 ± 42	0.63 ± 0.1	36.5 ± 23.5	71.2 ± 57.5	0.56 ± 0.1
October	4.8 ± 11.2	12.8 ± 9.7	0.74 ± 0.2	22.3 ± 14.3	45.8 ± 41.2	0.58 ± 0.2	29.3 ± 17.7	59.3 ± 50.5	0.57 ± 0.1
November	3.6 ±8.5	11.5 ± 8.5	0.74 ± 0.2	19.7 ± 12.1	39.3 ± 32.3	0.6 ± 0.2	27.5 ± 14.5	57.5 ± 41	0.54 ± 0.1
December	4.6 ± 7.7	14.9 ± 8.1	0.76 ± 0.2	21.7 ± 11.8	46.3 ± 35.7	0.56 ± 0.2	23.8 ± 12.9	52.2 ± 40.4	0.55 ± 0.16

**Fig. 4:**
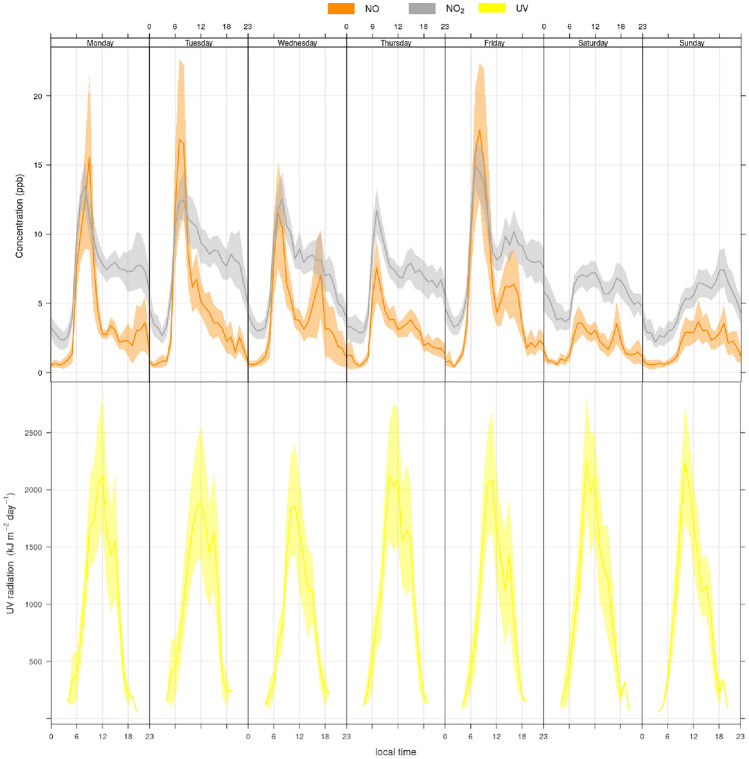
Weekly variability of hourly $\hbox {NO}_{2}$, NO and UV radiation over the whole period July to December at Roskildevej. The shading indicates a 75% confidence interval of the mean

The weekly pattern of hourly NO and $\hbox {NO}_{2}$ concentration at Roskildevej is presented in Fig. 4: daily mean $\hbox {NO}_{x}$ on weekdays resulted 22.7 (± 24.9) $\upmu \text {g}\,\text {m}^{-3}$, while on weekends $\hbox {NO}_{x}$ concentration decreases by 37% to 14.3 (± 12.2). The hourly average $\hbox {NO}_{2}$:$\hbox {NO}_{x}$ ratio during weekdays is 0.73 (± 0.2) and increases to 0.77 (± 0.1) on weekends, indicating a decrease in primary NO emissions from traffic, which might lead to an $\hbox {O}_{3}$ accumulation earlier in the day. Weekday-related variation shows higher NO and $\hbox {NO}_{2}$ accumulation on Fridays and could potentially be related to higher traffic intensity and less photodissociation of $\hbox {NO}_{2}$. A morning rush hour peak is observed around 7 to 8 AM local time (LT) and followed by a plateau lasting until 5 PM LT. Assuming local traffic as the major source of NO and primary $\hbox {NO}_{2}$, NO is quickly oxidised by ground level $\hbox {O}_{3}$ or hydrocarbon radicals to $\hbox {NO}_{2}$, thereby increasing $\hbox {NO}_{2}$ concentration. The midday plateau in NO and $\hbox {NO}_{2}$ levels is presumably a combination of maximum atmospheric mixing, along with photodissociation due to higher solar radiation and traffic emissions. As a result, the afternoon rush hour NO peak around 5-6 PM is less noticeable for all days, although present on Wednesdays and Fridays. $\hbox {NO}_{x}$ at Roskildevej exhibits a mild monthly variability, with a mean daily concentration ranging between 12.7 $\upmu \text {g}\,\text {m}^{-3}$ and 24.0 $\upmu \text {g}\,\text {m}^{-3}$ in July and October, respectively, and maximum daily $\hbox {NO}_{x}$ ranging between 22.7 $\upmu \text {g}\,\text {m}^{-3}$ and 75.5 $\upmu \text {g}\,\text {m}^{-3}$ in August and November, respectively; generally $\hbox {NO}_{x}$ increases in late Autumn and Winter, particularly in peak values (see Figs. 3 and 6), with September having the largest median.

The NO removal efficiency was calculated as in Folli et al. (2015) and resulted in $-0.17$ ± 1.27 after the comparison of the reference period and the PAP, i.e. when the maximum NO abatement is expected. The observed efficiency and its variability in $\hbox {NO}_{x}$ might be due to a few concurrent causes such as the SARS-CoV-2 restrictions, the summer holiday season, the meteorological conditions, as well as the active photocatalytic NOxOFF™ asphalt. In June, only a few activities in the public sector reopened, while in August most activities, education and workplaces reopened. The short length of reference period prior the photocatalytic application, is hampering an accurate direct estimate of the difference. However, looking at the $\hbox {NO}_{2}$:$\hbox {NO}_{x}$ ratio at Roskildevej there is an increases during the PAP period relative to the reference period, while no significant changes in this same ratio are observed at Jagtvej and HCA-B. This could indicate a decrease in NO levels despite the increase in traffic during PAP compared to the reference period.

**Fig. 5:**
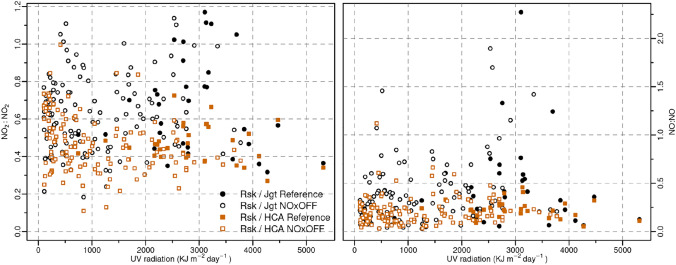
Ratio of daily mean $\hbox {NO}_{2}$ (left) and daily mean $\hbox {NO}_{2}$ (right) between Roskildevej and both Jagtvej and HCA-B, represented according to UV radiation, splitted between the Reference period (June 15–July 24) and the period with the photocatalytic surface

$\hbox {NO}_{x}$:$\hbox {NO}_{x}$ ratio comparison between Roskildevej and Jagtvej and HCA-B over the studied period are presented in Table 2. These ratios are used to minimise the influence by meteorology and SARS-CoV-2 restrictions on air pollution levels. However, Kruskal–Wallis and Wilcoxon–Mann–Whitney nonparametric statistical tests showed no statistically significant differences in $\hbox {NO}_{x}$:$\hbox {NO}_{x}$ ratio between the reference period and the PAP. A small decrease of $\hbox {NO}_{x}$ ratio for Roskildevej/Jagtvej is observed from October to December that could indicate a photocatalytic effect, however we have the lowest UV irradiance in this period. Daily mean $\hbox {NO}_{2}$:$\hbox {NO}_{2}$ and NO:NO ratio between Roskildevej and traffic stations in Copenhagen is plotted in Fig 5 against the UV radiation. Values from reference period are expected to be highest for NO, but no major differences are observed between the reference period and after NOxOFF™ application.

**Table 2:** Ratio of hourly $\hbox {NO}_{x}$ between Roskildevej and both Jagtvej and HCA-B at the UV peak time (12–14 UTC+1) during the Reference period (June 15–July 24) and the Photocatalytic Active Period (PAP)

Period	$$\hbox {NO}_{x}$$\$$\hbox {NO}_{x}$$	
	Roskildevej\Jagtvej	Roskildevej\HCA-B
Reference	0.6 ± 0.5	0.3 ± 0.1
PAP	0.6 ± 0.4	0.3 ± 0.2
September	0.7 ± 0.6	0.3 ± 0.3
October	0.5 ± 0.4	0.4 ± 0.3
November	0.5 ± 0.4	0.3 ± 0.2
December	0.5 ± 0.3	0.5 ± 0.3

Using the “Tunnel photo-red” model and street canyon variables and geometry, the upper limit $\hbox {NO}_{x}$ removal for the whole period after photocatalytic application was theoretically estimated to be 17.5%, assuming a street canyon effect and that the wind blows over the whole photocatalytic surface.

**Fig. 6:**
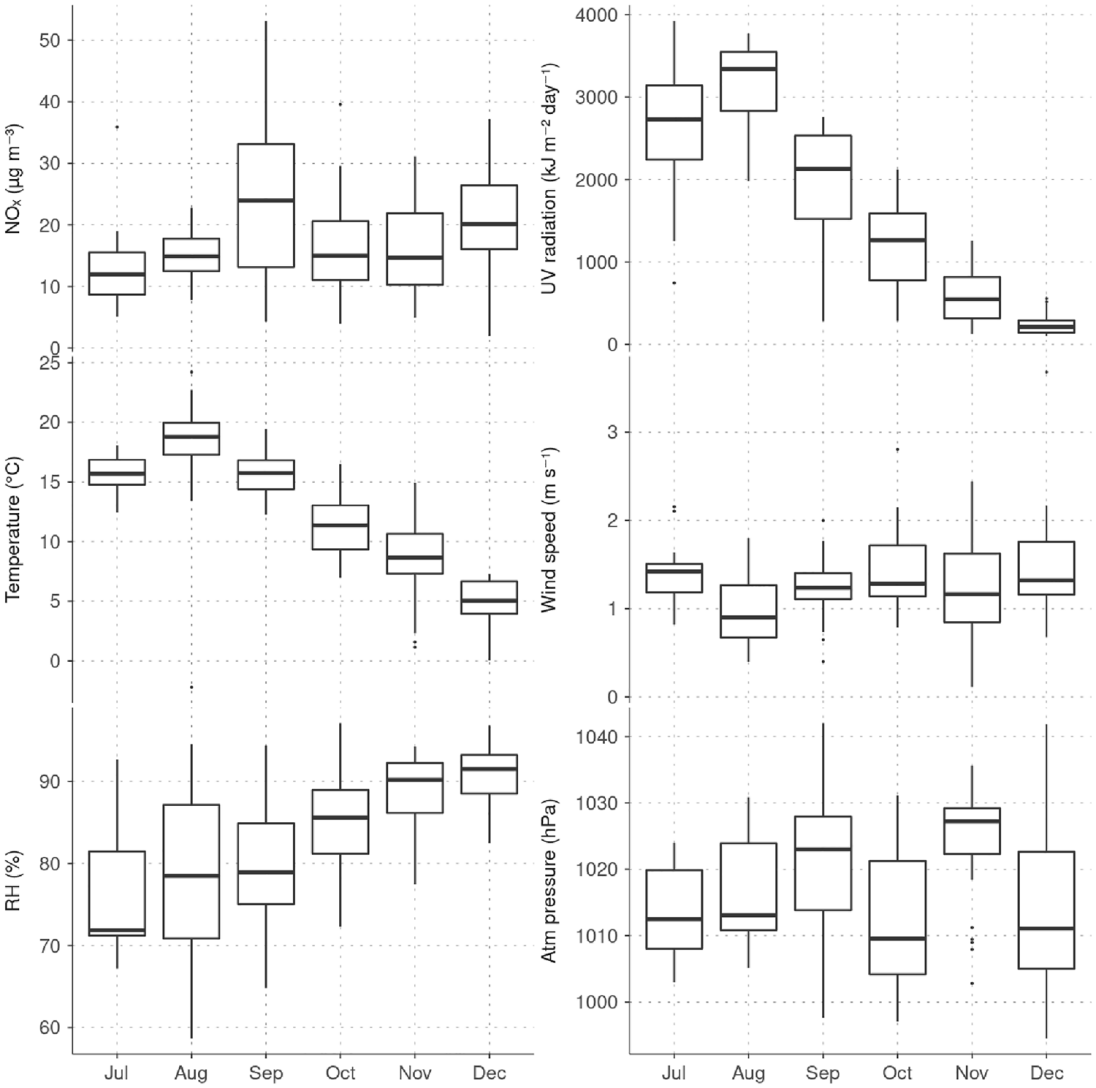
Monthly boxplot for daily $\hbox {NO}_{x}$, UV radiation, temperature, relative humidity, wind speed and surface pressure. The boxplot represents the median, lower twenty-fifth percentile and upper seventy-fifth percentile. Outliers are shown as dots

**Fig. 7:**
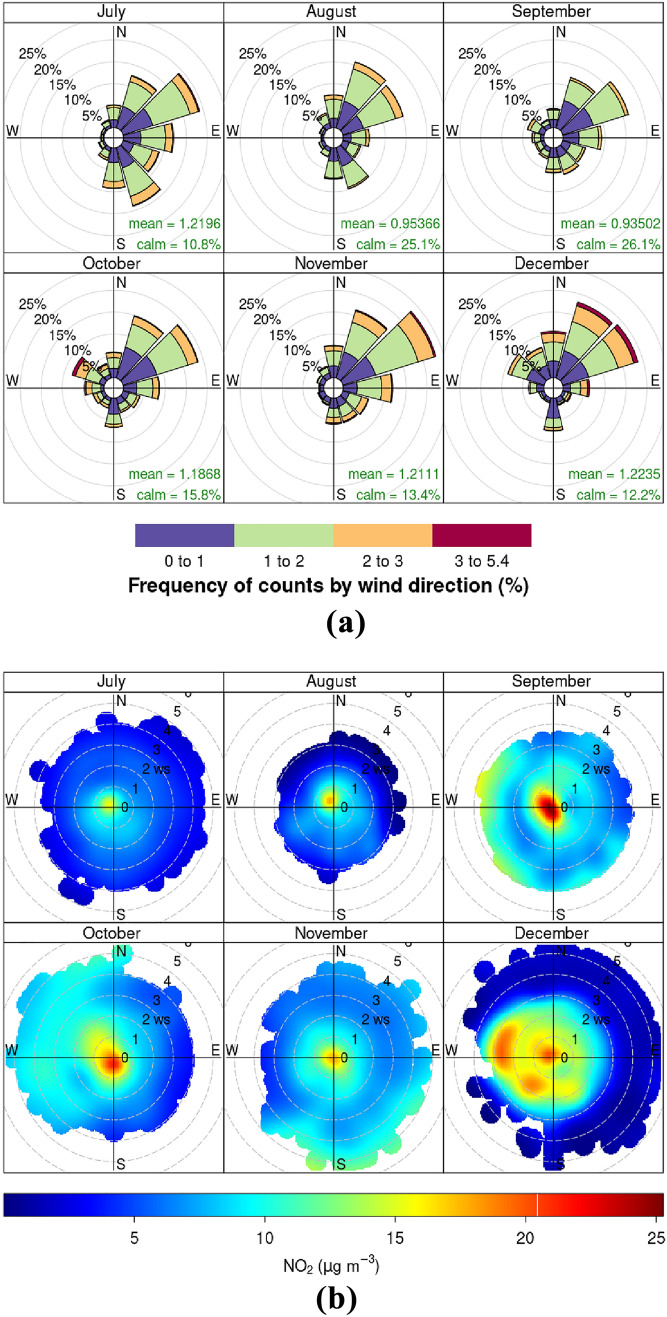
**a** Wind rose fragments and **b** bivariate polar plots for the period July to Dec., based on 1-min mean wind speed, wind direction, and $\hbox {NO}_{2}$ concentration

A comparison of monthly variations of $\hbox {NO}_{x}$ with other meteorological parameters measured at Roskildevej are shown in Fig. 6. Temperature is correlated with UV radiation and highest UV intensity is observed in August with a daily median of 312.2 W m$^{-2}$, while decreasing to 13.6 W m$^{-2}$ in December. The median solar radiation for the period after applied photocatalytic surface is 59.6 W m$^{-2}$ and, based on ERA5 reanalysis at its closest grid point to Roskildevej, the total mean UV radiation was estimated in 1349 kJ m$^{-2}$ day$^{-1}$. The average wind speed measured at Roskildevej was relatively constant throughout the campaign, with a median of only 1.3 m s$^{-1}$. Wind rose fragments of each month in Fig. 7a suggest, that wind is more frequently blowing from NE during the measurement period. The monthly bivariate polar plots for $\hbox {NO}_{2}$ in Fig. 7b provide an indication of the $\hbox {NO}_{2}$ mean concentration level (scaled by colour) associated to a specific wind direction and speed, expressed in radial coordinates (Uria-Tellaetxe and Carslaw 2014). The bivariate polar plots indicate that on average, when slow winds blow from SW-W, higher $\hbox {NO}_{2}$ occurs. The low wind speed typically indicates that dispersion is limited in the area which can cause accumulation of $\hbox {NO}_{2}$ close to the monitoring site. Moreover, E-NE winds are generally associated to a dilution of $\hbox {NO}_{2}$ at the site, while the less frequent W winds (occurring mainly in December) are associated with higher levels of $\hbox {NO}_{2}$. Therefore, the $\hbox {NO}_{2}$ levels at Roskildevej from July to December did not appear to be directly affected by the $\hbox {NO}_{2}$ plumes coming from Copenhagen city (NE direction). Possibly, this could be explained by the fact that the AWS is located at the southern side of the street, while the north side of the street is covered by a large, open green areas. Considering that the AWS is located at a low height, the polar plots could reveal also some micro-meteorological effects occurring in the urban environment. It could mean that the wind was recirculated, which highly depends on the size of the surrounding residential buildings, trees, etc. at the measurement site, potentially explaining the increase in $\hbox {NO}_{2}$ associated with moderate W winds in December. Other possible explanations could be a sudden event or the presence of a $\hbox {NO}_{2}$ source, which was not active in the previous months.

$\hbox {NO}_{2}$ concentration at the roadside highly depends on the number of vehicles, vehicle type, category, velocity and fuel among others. The daily traffic flow at Roskildevej is typically about 17.9k vehicles per day, although it is uncertain and challenging to determine precisely to what extent the traffic density has increased in the post-lockdown period (from June to December), and direct vehicle counts are not available for the investigated period. A SARS-CoV-2 pandemic mobility report using Google data revealed a 50% reduction of presence in workplaces during July 2020 compared to business-as-usual (pre-pandemic period) and a 25–30% drop from September to November 2020 (Google 2021).

**Fig. 8:**
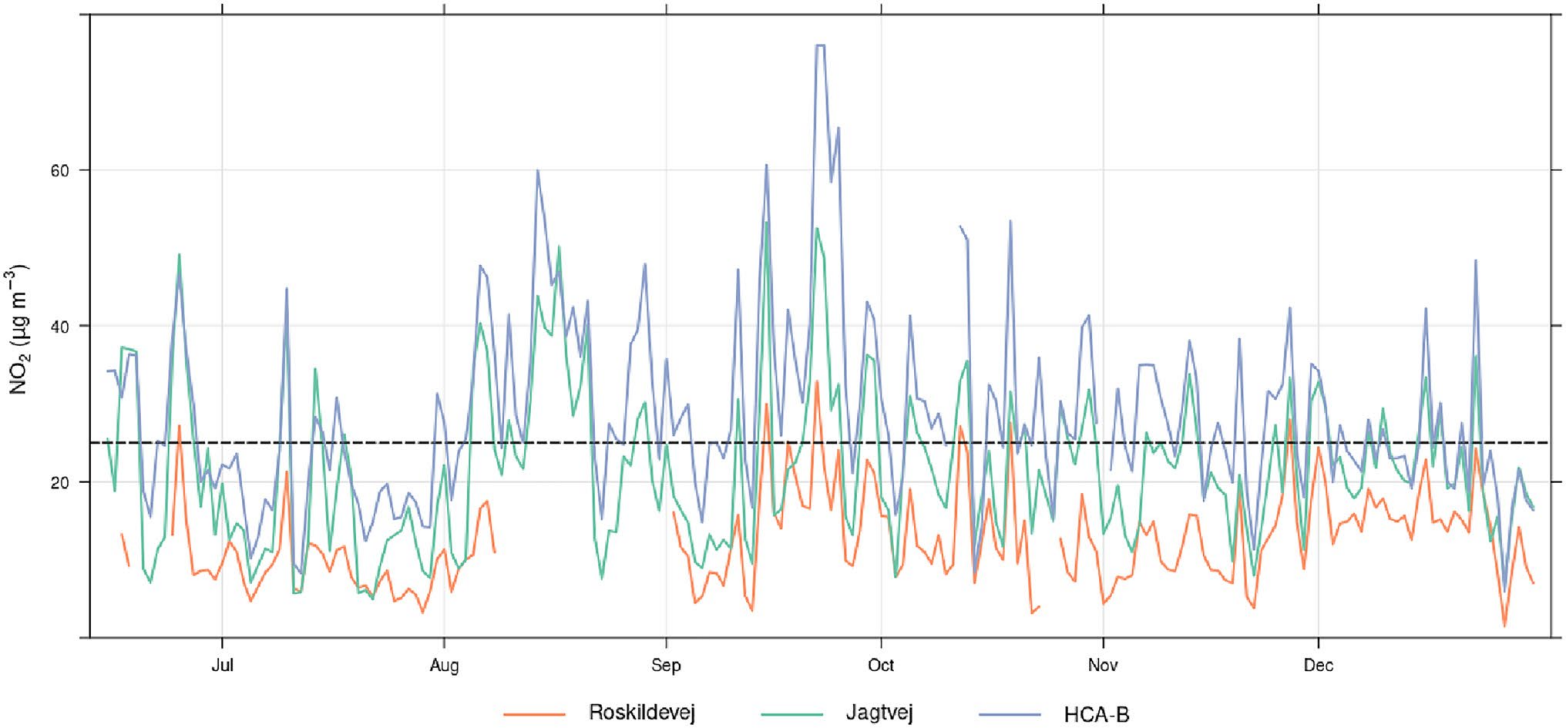
Daily averages of $\hbox {NO}_{2}$ in year 2020 measured at Roskildevej, Jagtvej, and HCA-B. Black horizontal line indicate the WHO AQ guideline of $\hbox {NO}_{2}$ daily limit value of 25 $\upmu \text {g}\,\text {m}^{-3}$

The monthly median $\hbox {NO}_{x}$ slightly increased in September and October and is likely associated with an increase in traffic compared to summer, but probably also due to a high pressure system lasting several days, associated to very low wind speeds (see Fig. 6), as shown by the concurrent increase in September also at Jagtvej and HCA-B (Fig. 8). In 2020, the $\hbox {NO}_{2}$ concentration at the two traffic stations HCA-B and Jagtvej, in the period after photocatalytic application, was measured to be 30.1 (± 18.5) $\upmu \text {g}\,\text {m}^{-3}$ and 22.3 (± 14.6) $\upmu \text {g}\,\text {m}^{-3}$. Furthermore, a Google streetview car (Google 2022) equipped with a CAPS $\hbox {NO}_{2}$ monitor (Aerodyne Research Inc, USA), estimated the yearly average $\hbox {NO}_{2}$ concentration at Roskildevej before SARS-CoV-2 lockdown, resulting in 24–29 $\upmu \text {g}\,\text {m}^{-3}$, consistently with the model projection to 2020 of $\hbox {NO}_{2}$ concentration at traffic sites of 24 $\upmu \text {g}\,\text {m}^{-3}$ in the Copenhagen area, including Frederiksberg.

Roskildevej data are compared with Jagtvej monitoring station to correct for the effect of Covid-19 restrictions on traffic and obtain a business-as-usual scenario at Roskildevej. The observed daily mean $\hbox {NO}_{2}$ concentration at Jagtvej in the period 2017–2019 with respect to 2020 decreased by 17% (± 92%) and was used to correct the mean $\hbox {NO}_{2}$ concentration at Roskildevej and compared to the air quality model projections based on COPERT V traffic emissions of 24 $\upmu \text {g}\,\text {m}^{-3}$ (0–27% uncertainty). The outcome of this comparison showed that $\hbox {NO}_{2}$ concentration was roughly estimated to decrease by 39% (± 38%), although this estimate is affected by high uncertainty. The correction factor for the impact of Covid-19 restrictions on air pollution levels did not explicitly account for the influence of meteorology, which also affects air pollution levels, although its influence was minimised by the comparison of $\hbox {NO}_{2}$ levels in the year 2020 (August to December) with the same months in the 3-year long period 2017–2019. Moreover, model uncertainties are also underestimated.

## Conclusion

Several studies showed how photocatalytic surfaces have a great potential for reducing $\hbox {NO}_{x}$ in urban areas, thus improving local air quality. The present study evaluates the effect of urban air quality improvement after the application of photocatalytic asphalt granulates (NOxOFF™) in an urban background site along Roskildevej, within the Frederiksberg municipality, an enclave within the Copenhagen municipality, Denmark. Several events hampered the analysis: the uncommon emission conditions because of to the restrictions due to the SARS-CoV-2 pandemic; the short reference period of observations prior the application of the asphalt; data gaps for $\hbox {NO}_{x}$. Therefore various methods were approached to evaluate the ability of the photocatalytic asphalt to reduce $\hbox {NO}_{x}$ levels: – a comparison of local $\hbox {NO}_{x}$ before and after the application of the asphalt, using both absolute levels and levels normalised by concurrent $\hbox {NO}_{x}$ at two other air quality monitoring sites in Copenhagen; – the application of a chemical kinetic model developed for street canyons with photocatalytic asphalts; – comparison with $\hbox {NO}_{2}$ model projections for Frederiksberg by air quality simulations based on street emission scenarios.

The NOxOFF™ capacity of removing ambient $\hbox {NO}_{2}$ at Roskildevej was roughly estimated by comparing observations with $\hbox {NO}_{2}$ model projection for year 2020 in Frederiksberg, based on air quality simulations with COPERT V street traffic emissions. The removal rate of $\hbox {NO}_{2}$ would hint to a reduction of 39% when compared with COPERT V emission model projections of 24 $\upmu \text {g}\,\text {m}^{-3}$ annual mean $\hbox {NO}_{2}$ (in agreement with Google car estimation), although this estimate is highly uncertain. The estimated value was corrected by 17% due to the difference in traffic density between the pre-pandemic years of 2017–2019 and 2020, as determined from a similar urban road named Jagtvej in Copenhagen. The removal capacity is considered very high compared to the theoretical upper limit $\hbox {NO}_{x}$ removal that was estimated to be 17.5% and the wide disparity between estimates is due to the significant amount of uncertainty involved.

The daily mean $\hbox {NO}_{2}$ concentration from July to December was 12.5 (± 5.9) $\upmu \text {g}\,\text {m}^{-3}$; although this cover only 6 months of the year, it is compliant with the 2030 clean-air Agenda of Frederiksberg municipality aiming to annual mean levels of $\hbox {NO}_{2}$ within 15 $\upmu \text {g}\,\text {m}^{-3}$ in urban background conditions.

## Data Availability

Air quality data were retrieved from DCE database, https://www2.dmu.dk. The dataset generated during the measurement campaign is not publicly available but is available from the authors upon request.
